# 

**Published:** 1912-05-18

**Authors:** 


### Appointments.

The following appointments have been made at the Manchester Royal Infirmary: Dr. Judson S. Bury, M.D., F.R.C.P., to be honorary consulting physician; Dr. E. M. Brockbank, M.D., F.R.C.P., senior honorary assistant physician, to be honorary physician; and Dr. Albert Ramsbottom, M.D., Ch.B., D.P.H. (Vict.), M.R.C.P. (London), to be honorary assistant physician.

### The Week's Novelties.

### THE MAXIPHONE.

This particular instrument for assisting deaf persons to carry on a conversation comfortably is constructed on an already familiar principle, but has certain features which give it a material advantage over many other instruments on the market. The first, and the most valuable one to those afflicted with deafness, is a simple arrangement whereby the intensity of the transmitted sounds can be varied to suit individual cases and special circumstances. It is a serious drawback when an otherwise helpful instrument cannot be made to accommodate itself to the varying loudness of the sounds which the patient wishes to hear. The Maxiphone has a regulating adjustment on the receiver, and in this direction is worthy of commendation. Naturally, there are also varying degrees of deafness, and this instrument allows of considerable range, so that progressive cases need not fear that the usefulness of the apparatus will be of only short duration. The Maxiphone Co., Ltd., of 92 Great Russell Street, W.C., will adjust or correct their instruments to suit requirements free of charge. The apparatus itself is neat and compact, allowing the user to carry it unobtrusively but conveniently. It consists of a small battery, a transmitter, and a receiver. The transmitter is attached in a second to the terminals of the battery by slip-on connections, so arranged that no mistake can be made, while the transmitter is easily held in the hand or may be attached to a head-band. The whole is enclosed in a neat case for carrying, and the cost is much less than that of several similar but older types; the battery costs a shilling for renewal. The efficacy of this instrument after a practical trial appears to be considerable, and more satisfactory in many ways than some others. No extravagant claims are made for it, but it may be confidently recommended as a useful aid for those afflicted with certain forms of deafness, and would undoubtedly spare those sufferers much harmful strain.

### OUR BUREAU OF INFORMATION.

### The Value of Accredited Correspondents.

Philanthropic attempts to lessen suffering are no longer isolated as they used to be. The contagion of pity has spread throughout the community, and those whose privilege it is to seek out the helpless no longer have to cope single-handed with the distresses they bring to light. The extent to which charity has become systematised by being shared among an ever-widening band of practical business workers sometimes awakes a fear that the essence of this good thing may disappear, crushed out of recognition under tabulated lists and reports of "cases." When we merely read of organised charitable efforts instead of taking part in them we get a dreary effect, as though these were not human lives which were at stake, but mere dried specimens in a museum, tabulated and placed on shelves. This is the outsider's view, and it extends to all philanthropic work systematically performed, not excluding that undertaken in hospitals and homes.

There can be no question that the only way to rouse and keep alive the passion of humanity is by actual association with those it is desired to help. It is a fine thing to write a cheque for some deserving institution. It is a finer thing to follow it up by personal service to the institution. It is an admirable thing to subscribe to a convalescent home, or send on to it invalids in need of good food and good air. But the subscriber only reaps half the benefit he might himself get from the exercise of his charity if he make no effort to get into personal touch with those he aims at helping. To preserve benevolence from becoming a matter of mere routine and to keep the charity cheques out of the category of those drawn for the poor-rate, the call is for contact with the needy. Classify them as we may they remain intensely individual. A hundred rules and ready-made methods of bestowing relief will prove insufficient to guide the almoner in the very first case which comes under notice. All the variety which human nature affords is at the disposal of the social inquirer. To plunge into the sea of destitution, which at a distance is tinged with the most hopeless monotony, is to become aware of a vast seething world of the most varied interest, in which common virtues and vices assume the most perplexing manifestations.

No sooner is the attempt made to supply information through such a centre as we have established in these pages than it becomes evident that without personal contact one with another many wants cannot be dealt with at all. To undertake ourselves journeys to every part of the kingdom on behalf of our correspondents is manifestly impossible. Yet in every part of the kingdom there arise from time to time matters which cannot be satisfactorily dealt with save after consultation with some one on the spot. We confidently make the appeal for workers to aid us in various localities by their advice, their local knowledge, and their experience of special forms of relief. It is the personal element in charity which can alone make the Bureau a source of strength to The Hospital readers.

### How to Join the Brotherhood of Service.

There are no formalities or difficulties connected with joining the Brotherhood. Readers are invited to send a stamped envelope for an application form for membership in order that their names may be inscribed among those whose wants will receive priority of attention and on whose willing co-operation the Bureau may count for information.

### Rules for Correspondents.

1. Every letter, on whatever subject, must be accompanied by the coupon to be cut from the back cover (inside page) of The Hospital, current issue.
2. Replies by post cannot be given, save under exceptional circumstances, at the Editor's discretion.
3. Special needs of every description relating to those who are sick or in difficulties are made known in these columns free of charge.
4. Letters from Approved Homes in reply to special needs published in the Bureau should state terms and full particulars, and be sent prepaid under cover to the Editor of the Bureau with coupon and name enclosed for identification.
5. Proprietors of homes which have not yet been entered on the List of Approved Homes, but have spare accommodation likely to suit special needs, are invited to write for an application form for registration. The fee for registration is 5s.
6. Applications relating to business or employment from correspondents in an independent position must be accompanied by a postal order for 5s.
7. All communications to be addressed to The Editor of The Hospital Bureau of Information, 28 Southampton Street, Strand, London, W.C.

### Hospital Management and Training.

### Training for Girl of Eighteen without Premium.

Dr. Jacobson.—The majority of children's hospitals fix the lowest entrance age for probationers at twenty, twenty-one, or twenty-two. When they are accepted at a lower age a small premium is usually charged to compensate for the amount of teaching and supervision required by young girls. The following hospitals, however, require no premium and take probationers at the age of eighteen or nineteen: Alexandra Hospital for Children with Hip Disease, Queen Square, Bloomsbury; the Derbyshire Hospital for Sick Children, Derby; Home and Infirmary for Sick Children, Sydenham Road, Sydenham, London; the Victoria Hospital for Sick Children, Hull; the Infants' Hospital, Vincent Square, London; Home for Women and Children, Leeds; St. Mary's Hospital for Women and Children, Plaistow, London, E. There is a steady demand for educated probationers, but your daughter would be well advised to wait till she is twenty or at least twenty-one.

### Needs and Helpers.

### Home of Rest for Gentlewomen.

"D. E. M.," Yorkshire.—If you will write making application to be admitted to the home, and forwarding the names and addresses of two references acquainted with your circumstances, we shall be happy to forward your application to the right quarter.

### Mild Mental Case.

"A. B. C."—We note that no further replies are needed for this case.

### Letter-Box.

"G. L. E."—We are unable to send the addresses of correspondents. A prepaid letter would be sent on.

